# Supplementary figures and images for: On the morphological variability of Ichniotherium tracks and evolution of locomotion in the sistergroup of amniotes
Source: PeerJ. 2018 Jan 31;6:e4346. doi: 10.7717/peerj.4346 (PMC5797465; doi:10.7717/peerj.4346)

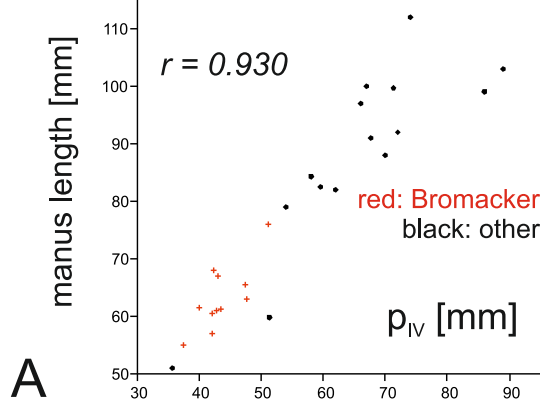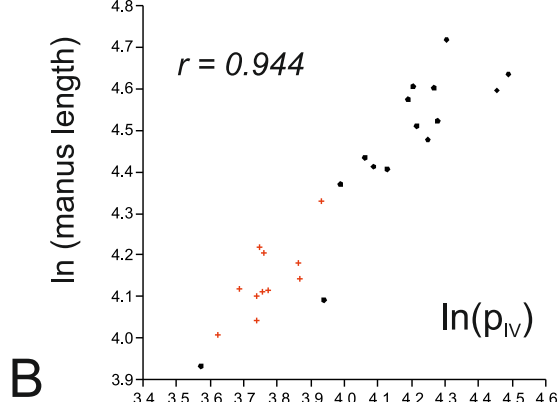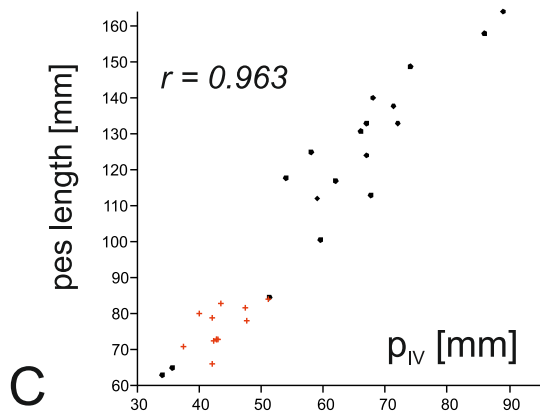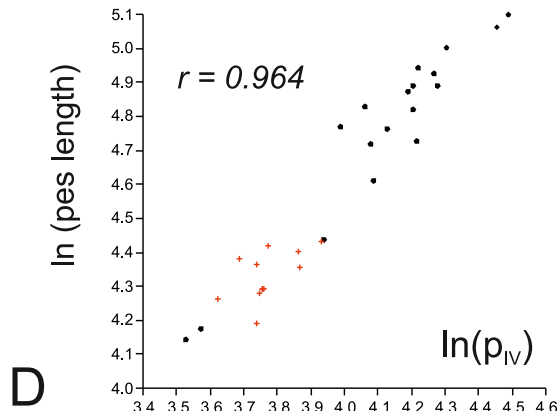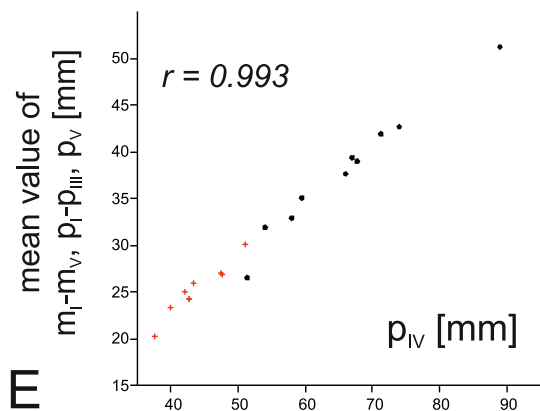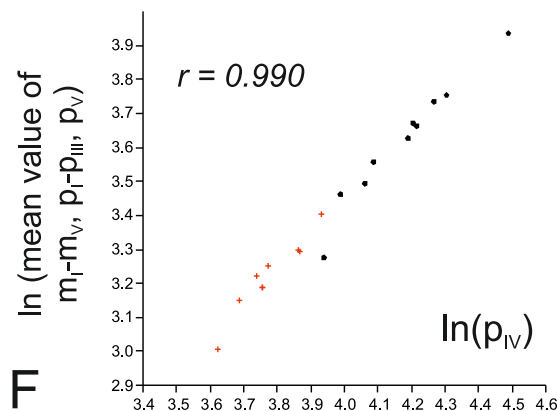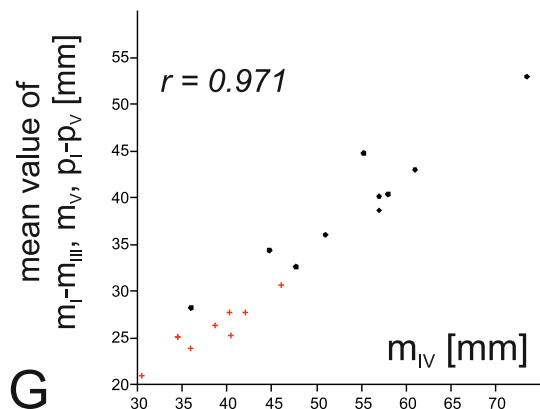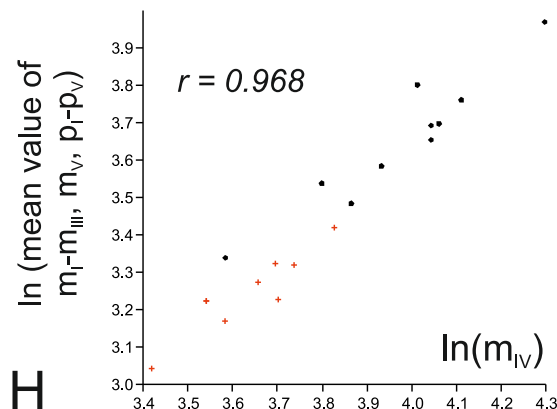

Supplement: Supplemental Information 5 — A–B, manus length vs. pedal digit length IV. C–D, pes length vs. pedal digit length IV. E–F, arithmetic mean of all other toe lengths vs. pedal digit length IV. G–H, arithmetic mean of all other toe lengths vs. manual digit length IV. Plots on the right side represent ln–ln plots of the same variable pairs depicted on the left side. [file peerj-06-4346-s005.pdf]
