# Supplementary material for: On the morphological variability of Ichniotherium tracks and evolution of locomotion in the sistergroup of amniotes
Source: PeerJ. 2018 Jan 31;6:e4346. doi: 10.7717/peerj.4346 (PMC5797465; doi:10.7717/peerj.4346)
Supplement: Supplemental Information 6 — The variable combinations and samples of the six depicted PCA results correspond to diagrams depicted in the manuscript: A, Biplot (PC1 vs. 2) for a PCA of four logarithmized manual toe ratios, corresponds to Fig. 12B. B, PCA result for the same data as in A but based on the correlation matrix. C, Biplot (PC1 vs. 2) for a PCA of four logarithmized pedal toe ratios, corresponds to Fig. 12A. D, PCA result for the same data as in C but based on the correlation matrix. E, Biplot (PC1 vs. 2) for a PCA of two logarithmizedlength ratios and four angle measures, corresponds to Fig. 13A. F, Biplot (PC1 vs. 2) for a PCA of two logarithmized length ratios and four angle measures, corresponds to Fig. 14C. [file peerj-06-4346-s006.pdf]

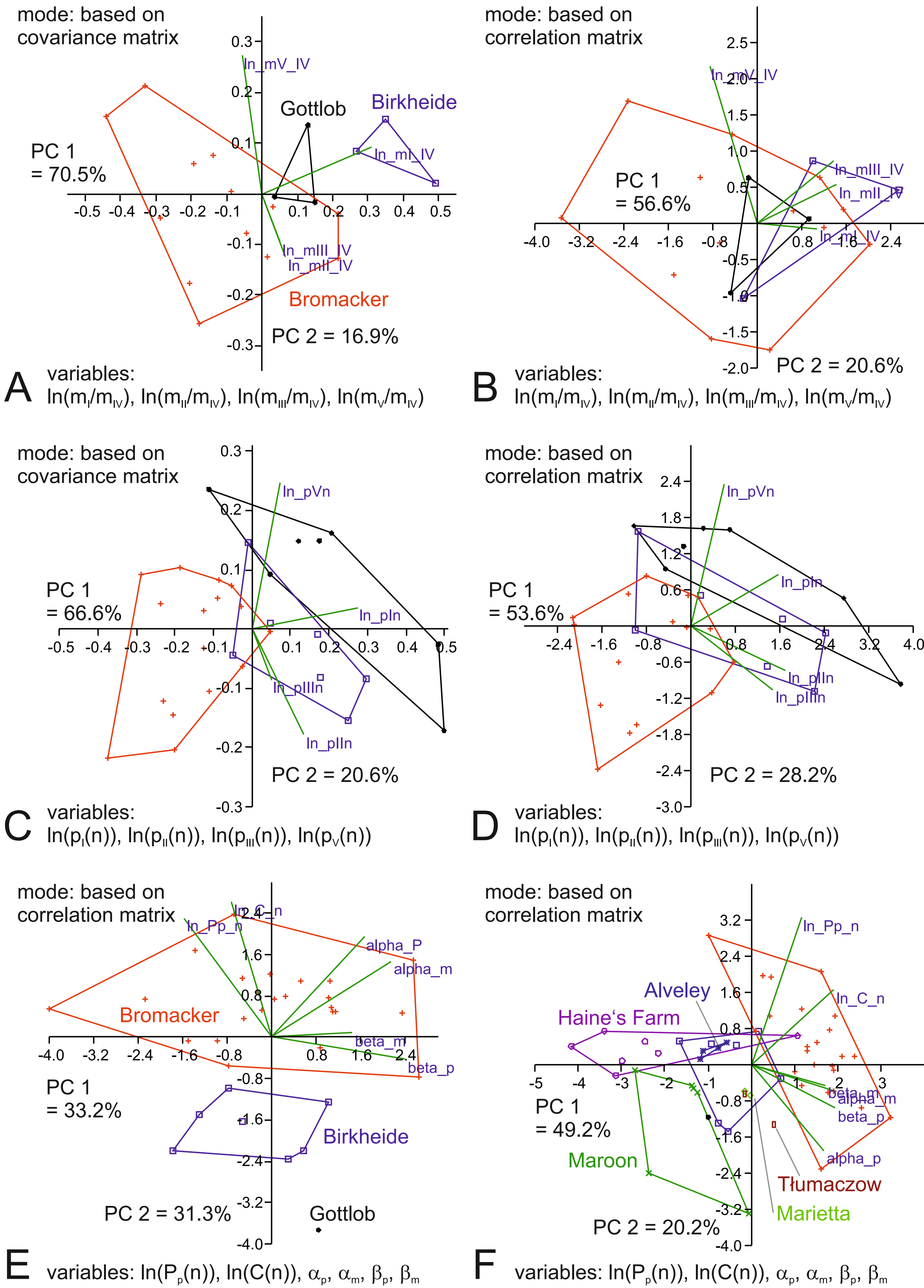

mode: based on covariance matrix
ln_mV_IV
Gottlob
Birkheide
ln_mI_IV
PC 1 = 70.5%
ln_mIII_IV
ln_mII_IV
Bromacker
PC 2 = 16.9%
A
variables: ln(mI/mIV), ln(mII/mIV), ln(mIII/mIV), ln(mV/mIV)
mode: based on correlation matrix
ln_mV_IV
ln_mIII_IV
ln_mII_IV
PC 1 = 56.6%
ln_mI_IV
PC 2 = 20.6%
B
variables: ln(mI/mIV), ln(mII/mIV), ln(mIII/mIV), ln(mV/mIV)
mode: based on covariance matrix
ln_pVn
PC 1 = 66.6%
ln_pIn
ln_pIIIn
ln_pIIn
PC 2 = 20.6%
C
variables: ln(pI(n)), ln(pII(n)), ln(pIII(n)), ln(pV(n))
mode: based on correlation matrix
ln_pVn
ln_pIn
PC 1 = 53.6%
ln_pIIn
ln_pIIIn
PC 2 = 28.2%
D
variables: ln(pI(n)), ln(pII(n)), ln(pIII(n)), ln(pV(n))
mode: based on correlation matrix
ln_C_n
ln_Pp_n
alpha_P
alpha_m
Bromacker
beta_m
beta_p
PC 1 = 33.2%
Birkheide
PC 2 = 31.3%
Gottlob
E
variables: ln(Pp(n)), ln(C(n)), αp, αm, βp, βm
mode: based on correlation matrix
ln_Pp_n
Alveley
ln_C_n
Haine's Farm
beta_m
alpha_m
beta_p
PC 1 = 49.2%
alpha_p
Maroon
Tłumaczow
Marietta
PC 2 = 20.2%
F
variables: ln(Pp(n)), ln(C(n)), αp, αm, βp, βm
